# A Robust Classifier to Distinguish Noise from fMRI Independent Components

**DOI:** 10.1371/journal.pone.0095493

**Published:** 2014-04-18

**Authors:** Vanessa Sochat, Kaustubh Supekar, Juan Bustillo, Vince Calhoun, Jessica A. Turner, Daniel L. Rubin

**Affiliations:** 1 Stanford Graduate Fellow, Graduate Program in Biomedical Informatics, Stanford University School of Medicine, Stanford, California, United States of America; 2 Department of Psychiatry & Behavioral Sciences, Stanford University School of Medicine, Stanford, California, United States of America; 3 Department of Radiology, Stanford University School of Medicine, Stanford, California, United States of America; 4 The Mind Research Network, Albuquerque, New Mexico, United States of America; 5 Department of Psychiatry, University of New Mexico, Albuquerque, New Mexico, United States of America; 6 Georgia State University, Department of Psychology and Neuroscience Institute, Atlanta, Georgia, United States of America; Medical University of Vienna, Austria

## Abstract

Analyzing Functional Magnetic Resonance Imaging (fMRI) of resting brains to determine the spatial location and activity of intrinsic brain networks–a novel and burgeoning research field–is limited by the lack of ground truth and the tendency of analyses to overfit the data. Independent Component Analysis (ICA) is commonly used to separate the data into signal and Gaussian noise components, and then map these components on to spatial networks. Identifying noise from this data, however, is a tedious process that has proven hard to automate, particularly when data from different institutions, subjects, and scanners is used. Here we present an automated method to delineate noisy independent components in ICA using a data-driven infrastructure that queries a database of 246 spatial and temporal features to discover a computational signature of different types of noise. We evaluated the performance of our method to detect noisy components from healthy control fMRI (sensitivity = 0.91, specificity = 0.82, cross validation accuracy (CVA) = 0.87, area under the curve (AUC) = 0.93), and demonstrate its generalizability by showing equivalent performance on (1) an age- and scanner-matched cohort of schizophrenia patients from the same institution (sensitivity = 0.89, specificity = 0.83, CVA = 0.86), (2) an age-matched cohort on an equivalent scanner from a different institution (sensitivity = 0.88, specificity = 0.88, CVA = 0.88), and (3) an age-matched cohort on a different scanner from a different institution (sensitivity = 0.72, specificity = 0.92, CVA = 0.79). We additionally compare our approach with a recently published method [1]. Our results suggest that our method is robust to noise variations due to population as well as scanner differences, thereby making it well suited to the goal of automatically distinguishing noise from functional networks to enable investigation of human brain function.

## Introduction

It is now well established that knowledge of functional brain networks is fundamental to understanding how the human brain produces cognition. In recent years, resting-state fMRI (rsfMRI) has emerged as a powerful tool for studying functional brain networks [2]–[5]. Resting-state fMRI measures the spontaneous, synchronized fluctuations in the BOLD signal involved with information processing [6] and general maintenance and coordination of functional networks [7]–[9]. It allows examination of the functional organization of the brain outside of the demands of a particular task [10], [11], making it ideal for the study of functional brain networks in a wide range of ages and clinical populations [12]–[18]. Critically, resting-state fMRI can give valuable insight to normal and atypical development [19], [20] as well as disorder-specific aberrancy [21].

Although there is a general consensus about the importance of rsfMRI for investigating human brain function, its potential has not been fully exploited due to several reasons. Chief among them is the issue of recognizing and removing noise inherent in rsfMRI data [22], [23]. There are a significant amount of physiological, respiratory, and mechanical artifacts [24]–[26]. Typical strategies for dealing with such noise include statistically adjusting for spikes in data, removing time-points entirely, and filtering out noise based on a global metric such as percentage of high frequency signal. This task of detecting noise is challenging due to the complex mixing of artifact and physiological signal; thus, researchers have used independent component analysis (ICA) [27] as a solution [28], [29]. Mckeown et.al first applied ICA to fMRI with simulated noise, and later addressed the ability of ICA to separate the signals, and to reliably interpret and reproduce these signals [30], [31]. In this early work, it was clear that a standard for what constitutes noise (or equivalently, what comprises a functional brain network) would be needed; however, due to variability in datasets, this has proven to be a challenge.

To address this problem of identifying ICA components that represent noise, researchers first relied on the same approaches used to remove it from rsfMRI data, including filtering based on simple metrics of the time-course, as well as manual annotation and template matching [32]–[34]. More recently, researchers have developed more sophisticated learning algorithms to group independent components [1], [29], [34]–[37]. While the performance of these approaches is promising, more work is needed to employ flexible feature selection that makes no *a-priori* assumptions about the signature of noise. A recent method contributes to this goal by employing ensemble learning [38], and we extend this flexible approach by (1) addressing this challenge through a novel strategy, (2) going further to include a formal performance evaluation through direct comparison with other published methods, and (3) testing our method on more than two datasets that cross institutions, scanners, and disorder types. The automatic filtration of components is critical for accurately identifying and removing ICA components that represent noise from the ever-growing publicly available resting-fMRI datasets that contain data from different populations collected at different sites [11], [39]–[41].

In this paper, we describe a novel approach to automatically identify those ICA components derived from rsfMRI datasets that represent noise. We use ICA analysis to extract functional networks and noise, followed by a supervised learning algorithm to define identifying features of the noise. We make these features and methods available in a publicly accessible database (http://www.vbmis.com/bmi/noisecloud). We demonstrate the utility and robustness of this method by developing models of noise using a set of healthy control rsfMRI datasets, and then extending the models to age- and scanner-matched cohorts of (1) patients from the same institution with a neuropsychiatric disorder, (2) healthy control datasets acquired on an equivalent scanner from a different institution, and (3) healthy control datasets acquired on a different scanner from a different institution. We also develop a model to detect noisy components in a group decomposition of the original healthy control rsfMRI to demonstrate application of our methods to group ICA, as well as formally compare our method to a recently-published approach [1]. Our approach has the potential to become an efficient and useful tool for filtering large rsfMRI datasets to make possible large-scale, data-driven neuroscience research.

## Materials and Methods

### 2.1 Overview of Our Approach

**Figure 1:**
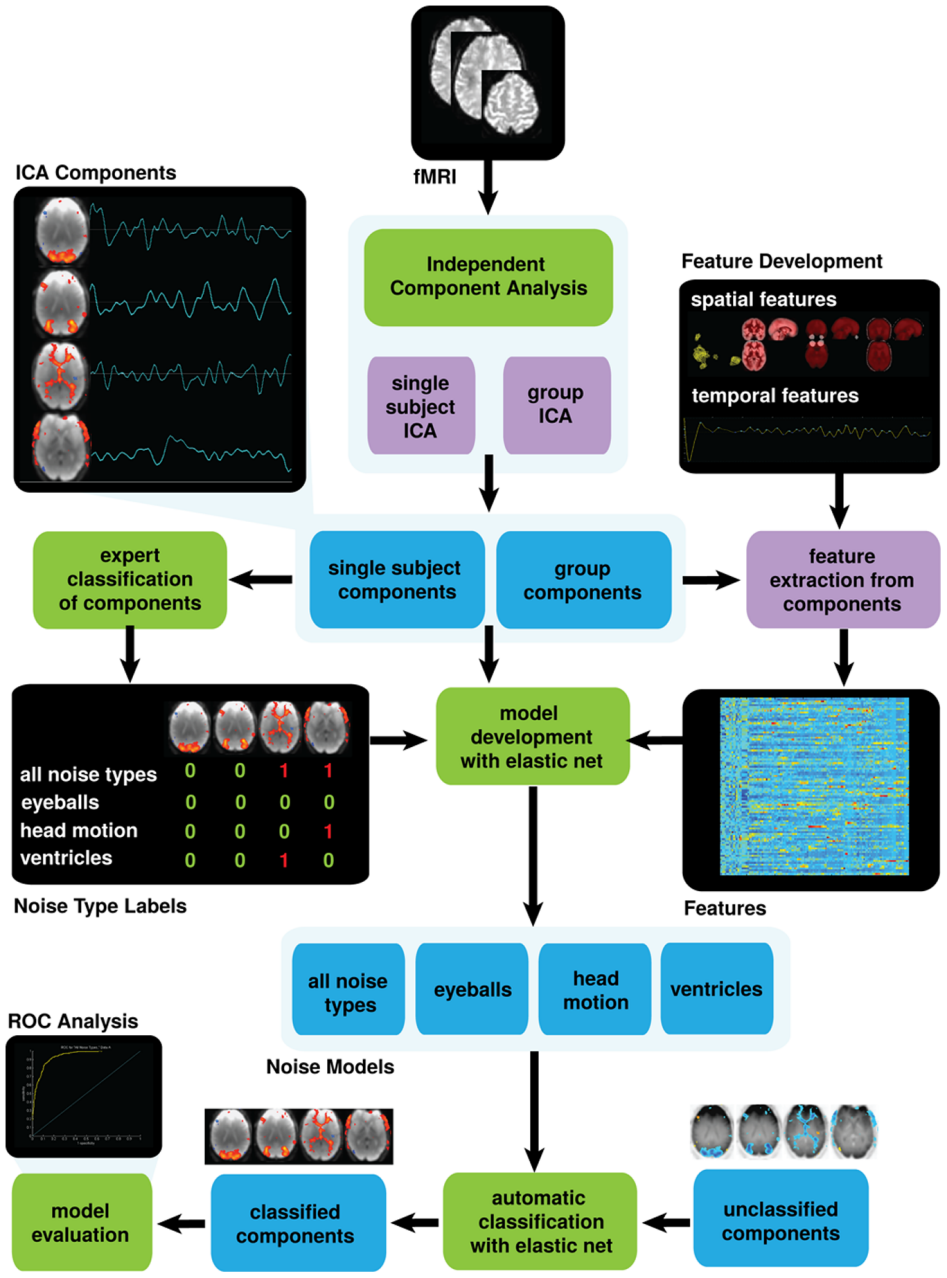
Overview of our approach. We preprocessed our four fMRI datasets (fMRI), and used independent component analysis to extract both individual and group ICA components, indicated at the top of the figure. We concurrently developed comprehensive spatial and temporal features to describe our components (feature extraction from components), and manually labeled our components as noise or not noise (expert classification of components). Both features and labels allowed for the use of sparse logistic regression to build seven models (model development with elastic net) of components from individual ICA, and one model of components from group ICA (Noise Models: all noise types, eyeballs, head motion, and ventricles). The four successful individual ICA models and group model (N = 5) were then evaluated. This evaluation included using unclassified components from external data (automatic classification with elastic net) to predict component types (classified components) and ROC curve analysis. Summarized results are indicated in Table 1.

An overview of our approach is provided in **Figure 1**. The goal of our methodology is to enable automatic identification of noisy ICA components by assessing the spatial and temporal features of these components. We adopt a machine learning approach to this task which uses sparse logistic regression with elastic net regularization to both build a model and to select relevant features. We first select four datasets with differences in the institution where the data was acquired, population, and scanner type (see *2.2 Study data*). We use independent component analysis to extract both individual and group ICA components (see *2.3 Independent component* analysis), and develop a database of comprehensive spatial and temporal features for the classifier to choose from to describe these components (see *2.4 Characterizing independent components using spatial and temporal features*). We then use sparse logistic regression to build seven models of components from individual ICA, and one model of components from group ICA (see *2.5 Distinguishing noise-related from network-related components using sparse logistic regression*) to demonstrate that this approach is advantageous in being able to build custom filters for different component types. The successful models concurrently provide human-interpretable spatial and temporal features that distinguish each component subtype with weights that reflect the strength of the contribution of each feature in the model. This particular quality of our method is essential in that we are able to computationally and semantically characterize components. Finally, we evaluate our approach by applying it to datasets across different institutions, scanners, and subject populations, and by comparing it with a recently published method (see 2.6 Evaluation).

### 2.2 Study Data

An overview of the data used in our study is shown in **Table 1**. We compiled 4 datasets that are used to build and to evaluate our methods.

**Table 1:** An individual and group model of “all noise types” (M1)(M5) was built using Data A, to be tested on three other datasets, Data B, C, and D.

Model	Labels	Individual/Group	Training Data	Testing Data
**M1**	All Noise Types	Individual	Data A	Data A, Data B, Data C, Data D
**M2**	Eyeballs	Individual	Data A	Data A, Data B
**M3**	Head Motion	Individual	Data A	Data A, Data B
**M4**	Ventricles	Individual	Data A	Data A, Data B
**M5**	All Noise Types	Group	Data A, Data B	Data A, Data B
**M6**	Primary Visual Cortex	Individual	Data A	Model not successful
**M7**	Parieto-occipital Cortex	Individual	Data A	Model not successful
**M8**	Ventral Primary Somatosensory Cortex	Individual	Data A	Model not successful

Specific noise types (M2)(M3)(M4) were successfully built with Data A, and then extended to Data B. Models of functional networks (M6)(M7)(M8) were not successful, and were not extended to other datasets. Ten-fold cross validation was used for evaluation of all models.

*Data A:* We obtained an existing dataset of rsfMRI for 29 healthy controls (mean age 29.48 years, 16 Male/13 Female) from the Mind Research Network [42]. Data were motion-corrected, spatially smoothed with a 6 mm full width at half-maximum Gaussian kernel, bandpass filtered (0.008 to 0.1 Hz) and spatially normalized into the standard Montreal Neurological Atlas Space in preparation for probabilistic ICA [43]. These data were used to build our initial model of noise in individual ICA to be extended to other data, and combined with Data B to build a model of noise for group ICA components.

*Data B:* This comprised a dataset with 24 individuals with Schizophrenia acquired with the same scanner and pulse sequences (mean age 35.125, 21 Male/3 Female) [44]. Schizophrenia was chosen because significant differences in connectivity of functional brain networks have been shown to exist [18]. Data were motion corrected, spatially smoothed, bandpass filtered, and spatially normalized. These data were used to test our initial model of noise in individual ICA components, and combined with data A to test a model of noise for group ICA components.

*Data C:* This comprised rsfMRI data from 24 healthy controls from the NKI Rockland resource [45]. Data C was used as a secondary testing of the model on data from a different institution and equivalent scanner. These data were processed equivalently to Data A and B, and individual ICA components extracted to test our initial model of noise.

*Data D:* These data were acquired from a different institution and a different scanner and was provided by the first release from the Human Connectome Database. To maintain anonymity, ages are provided in ranges (16 Male/13 Female datasets, 17 in range of 26–30, 11 in range of 31–35, and 1 in range of 22–25). Data were processed equivalently to all other datasets. This data collection and sharing was provided by the MGH-UCLA Human Connectome Project (HCP; Principal Investigators: Bruce Rosen, M.D., Ph.D., Arthur W. Toga, Ph.D., Van J. Weeden, MD). These data were used for the last test of our initial model of noise in individual ICA.

### 2.3 Independent Component Analysis

Independent Component Analysis, performed with MELODIC (Multivariate Exploratory Linear Decomposition into Independent Components) Version 3.10, part of FSL (FMRIB's Software Library), decomposed each of the individual datasets into independent components [43]. We allowed the software to employ automatic order selection, selecting the number of components to extract by using the Laplace approximation to the Bayesian evidence of the model order (Minka, 2000; Beckmann, 2004), and used the fast fixed-point-algorithm (fastICA), also built into MELODIC [46]–[48]. The fastICA approach finds the weight matrix (w) to solve for the independent components by iteratively maximizing the non-Gaussianity of the projection of the weights onto the observed data. This step in the analysis resulted in 880, 638, 1658, and 681 total components for each of Data A, B, C, and D, respectively.

Group independent component analysis was performed with MELODIC by doing a temporal concatenation with automatic dimensionality estimation for each of Data A and Data B, resulting in 25 and 19 components for each of Data A and B, respectively. These components were combined to build and test a model of group noise, an important task as group components are also commonly used in analysis of functional networks [17], [49], [50]. Additionally, the consolidation of data into a much smaller number of components allowed for the development of a set of noise labels curated by more than one expert.

### 2.4 Characterizing Independent Components using Spatial and Temporal Features

A total of 246 spatial and temporal features and automatic extraction methods, some based on current literature [32], [33], [37], [51], [52], and some novel, were developed, and features were extracted from all components. All features and extraction scripts are publicly available (http://www.vbmis.com/bmi/noisecloud). Spatial features included voxel counts for each of the 116 regions of the Automated Anatomical Labeling (AAL) atlas, metrics to describe the distribution of Z-score values of the spatial map (kurtosis, skewness, spatial entropy), clustering metrics (average distance between 10 most highly connected node pairs, minimum and maximum cluster sizes), mirror-likeness of a component (percent overlap of one hemisphere’s voxels reflected onto the other hemisphere), and region and tissue-specific activation percentages (white, gray, cerebral spinal fluid (CSF), eyeballs, edges, midbrain, skull, ventricles, cerebellum, spinal cord) [53]. Temporal features included equivalent metrics to describe the temporal time course (kurtosis, skewness, temporal entropy, mean response over time), percentage of high frequency energy, average number of local maximums and local minimums, average distances between local maximums and minimums, average and biggest jump from a minimum to a maximum value, power in multiple bands (0–0.008 Hz, 0.008–0.02 Hz, 0.02–0.05 Hz, 0.05–0.1 Hz, 0.1–0.25 Hz), autocorrelation (one through five lag), power spectral densities, and dynamic ranges and counts (the difference between peak power or counts and minimum or maximum power at frequencies to the right and left of the peak). A complete overview of spatial and temporal features is included in **Table S2**. Features are not biased by preprocessing parameters (for example, we include the band 0–0.008 despite bandpass filtering our data) to ensure extendibility to data processed with different strategies. Software to query the database and automatically extract features was developed, and employed to characterize all individual (Data A, B, C, and D) and group components (each of groups Data A and B).

### 2.5 Distinguishing Noise-related from Network-related Components using Sparse Logistic Regression

We developed an automated classifier to detect noisy rsfMRI components using logistic regression with the elastic net penalty, a cost function added to the optimization step of regression [54]. For 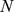 as the number of components to classify, 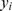 as a vector of class labels, 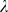 and 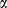 as parameters determined by cross validation and grid search (explained later), 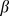 as the vector of weights to optimize, and 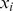 a feature matrix with 246 spatial and temporal features (columns) for each of 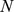 components, the elastic net optimization problem is defined as follows:
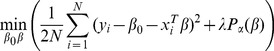
(A)where
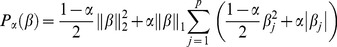
(B)


The first term in Equation **A** can be recognized as the least squares optimization technique, and the second is an additional penalty term, defined in Equation **B**. This penalty term is a weighted sum of (1) the 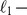 norm, which enforces the sparsity of the solution, and (2) the square of the 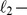 norm of the coefficients 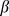 which selects groups of correlated features that are not known a-priori [55]. This technique employs intelligent feature selection by way of fluctuating between the Least Absolute Shrinkage and Selection Operator (LASSO), and the ridge regression penalty [56], [57]. The term alpha (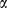) allows this fluctuation between LASSO (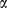 = 1) and ridge regression (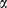 = 0). Ridge regression is suited to shrink the coefficients of correlated predictors toward one another, making it ideal for many predictors with non-zero coefficients [58]. The LASSO is more suited to choose one predictor and disregard the rest, by setting a small subset of predictors to have large coefficients and the rest to have coefficients close to zero. Combining the two (the elastic net) allows for intelligent feature selection by fine tuning the degree of sparsity, controlled by the tuning parameter alpha. A second parameter lambda (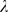) controls the sparsity and ridge regression simultaneously. By selecting these parameters via maximization of cross validated accuracy, the ideal threshold (meaning the number of non-zero coefficients (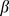) and features used to build the model) is automatically determined. This intelligent feature selection is an ideal strategy for this application, as it selects the minimum feature set from a much larger set to maximize the performance of the classifier, and one can quickly select a custom set and number for each component type.

We optimized the value of these parameters by finding the combination of values that maximized the 10-fold cross validation accuracy for each component type. During this optimization, alpha is varied ranging from 0 (produces a ridge regression solution, meaning that betas of correlated variables are made equal) to 1 (produces most sparse solution). Optimizing the weights in this iterative procedure also folds feature selection into one step, meaning that this machine learning method performs both feature selection and classification during the process of creating the model. The classifier takes as input a normalized set of spatial and temporal features describing each component, and ultimately produces, for each component, an output value between 0 and 1 that is typically thresholded at 0.5 to determine a binary decision (0 or 1) indicating whether or not a component represents noise (or the label of interest). The spatial and temporal features with non-zero coefficients (the selected features) represent a computational “signature” to describe the component type. The final classifier is constructed using these non-zero weights, to be used to predict the class of a novel component. To make a prediction, 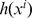, we are interested in the probability of a noisy component given the selected features, 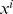 a subset of the entire set 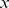, as determined by our subset of non-zero coefficients, 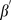 selected by the elastic net. The selected parameters are applied to a new data, 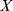, using the logistic function, shown in Equation **C**:
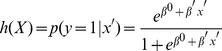
(C)


The output is between 0 (not noise) and 1 (noise), which is thresholded to produce a binary class label. The choice of threshold comes by way of receiver operator characteristic (ROC) curve analysis, where we select a threshold that maximizes overall accuracy, discussed in 2.6.5 *Evaluation of classifier performance*.

### 2.6 Evaluation

#### 2.6.1 Overview of our evaluation

**Figure 2:**
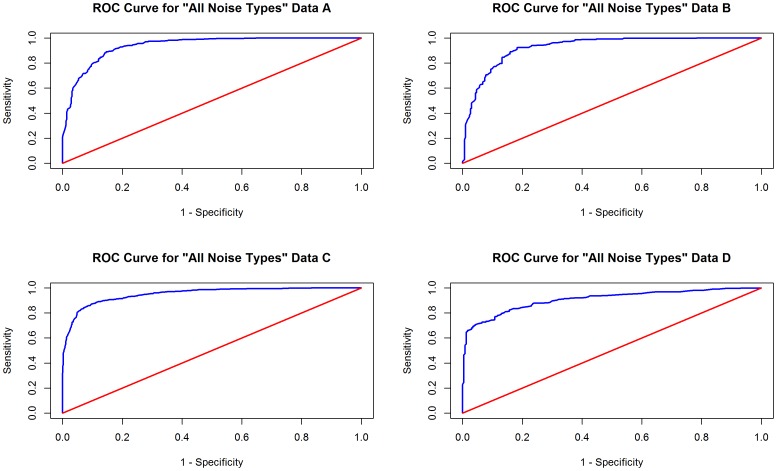
ROC analysis for “All Noise Types” Models. ROC Analysis of the classifiers trained and tested with ten-fold cross validation on Data A, B (same institution, different population), C (different institution, same scanner), and D (different institution, different scanner) for the “all noise types” models. The red line represents performance of a classifier that does no better than random chance.

To demonstrate the utility and robustness of this method for automatically characterizing and identifying noisy components, we first developed a “ground truth” by manual annotation of ICA components in rsfMRI data (see *2.6.2 Human curation of independent components*) and then built 7 models of different kinds of components, including one model of all noise types (see *2.6.3 Building models of component types).* Finally, we tested these models with data from different institutions and scanner types (see *2.6.4 Testing models of individual and group noise*). We evaluated our classifiers’ performance (see 2.6.5 *Evaluation of classifier performance*), and we compare that with results obtained with a recently-published method (see *2.6.6 Comparison with a recently developed method*). A summary of our evaluation approach is included in Figure 2.

#### 2.6.2 Human curation of independent components

**Figure 3:**
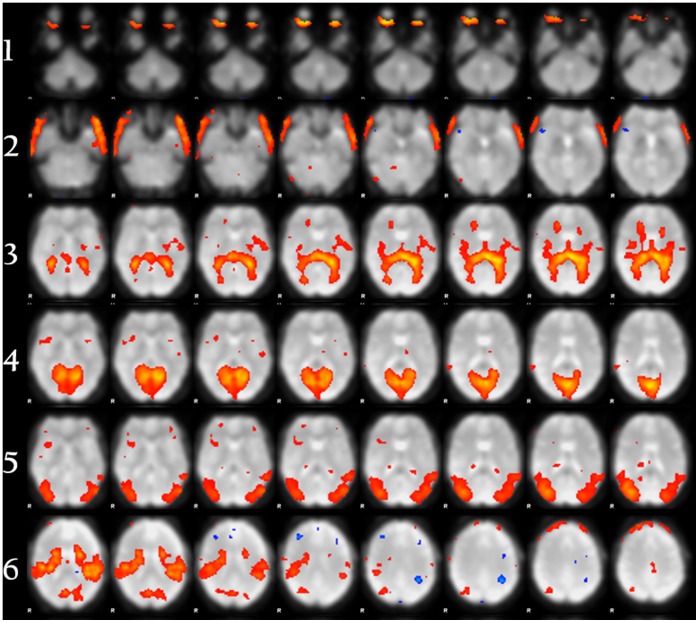
Component types. Representative spatial maps for noise and functional network subtypes used in the study including (1) eyeballs, (2) head motion, (3) ventricles, (4) primary visual cortex, (5) parieto-occipital cortex, and (6) ventral primary somatosensory cortex. For the “all noise types” models (not illustrated), criteria included signal outside of gray matter, high frequency time-courses, and the presence of rings or stripes that represent motion or scanner artifact.

We established a benchmark for validating our methods to identify noisy ICA components in two ways. First, a single expert reader (VS) carefully evaluated the components obtained from cases in Data A and B using a custom tool in Matlab and provided labels for seven specific types of ICA components, including noise subtypes (comprehensive noise “all noise types”, eyeballs, head motion, and ventricles), and functional network subtypes (primary visual cortex, parieto-occipital cortex, and ventral primary somatosensory cortex) across the 880 and 638 components (10,626 evaluations). This subset of component types was selected for annotation based on the ease of manual identification using the spatial map and time course. General indicators of noise used to create the labels included signal outside of gray matter (in ventricles, white matter, CSF, skull or surrounding tissue), high frequency time-courses, and the presence of rings or stripes that represent motion. The reader also provided labels for a standard that encompasses “all noise types” for Data C and D (2,339 evaluations). Representative spatial maps for noise and functional network subtypes are detailed in **Figure 3**.

Our second goal was to establish a benchmark for noise based on more than one expert reader. Since it would have been a formidable task to have multiple experts evaluate all individual component labels, we used multiple readers to assess the group components (requiring only 44 assessments per curator). Three expert readers undertook this task (VS, KS, CA). Each reader provided one set of labels for group components extracted from the group decompositions of Data A and B using a secure web interface (132 evaluations). The interface showed the equivalent spatial maps and component signals to the readers, who were instructed to label each component as “not noise” or “noise.” Any disagreement among the experts in the type of component was resolved through consensus.

#### 2.6.3 Building models of component types

We built models using both individual and group ICA decompositions to demonstrate the extendibility of our method to both. For individual ICA, a set of healthy control rsfMRI (Data A) was used to build seven models encompassing different kinds of noise and functional networks (all noise types, eyeballs, head motion, ventricles, primary visual cortex, parieto-occipital cortex, and ventral primary sensorimotor cortex). For group ICA, separate group ICA decompositions were done for Data A and Data B to create a set of combined healthy control and disorder-specific components, and a model encompassing “all noise types” was built using these combined group components.

#### 2.6.4. Testing models of individual and group noise

We tested each of our seven models described above encompassing different kinds of noise and functional networks with cross validation using the same healthy control (Data A). To test the generalizability of our methods and the extendibility of our models, we tested the four successful models from this set (all noise types, eyeballs, head motion, and ventricles) in an age- and scanner-matched cohort of patients from the same institution with a neuropsychiatric disorder (Data B). Finally, we extended one of the models (all noise types) to healthy control datasets acquired on an equivalent scanner from a different institution (Data C), and healthy control datasets acquired on a different scanner from a different institution (Data D). For evaluation of the method applied to group decomposition, a separate model was built and tested using the group temporal concatenation of Data A (25 components) combined with the group temporal concatenation of Data B (19 components). Details of how the testing was carried out and evaluated are given in Section 2.6.5.

#### 2.6.5 Evaluation of classifier performance

For each classifier, the output is a set of weights corresponding to the contribution of each of the 246 spatial and temporal features in the model. A weight of zero indicates that a particular feature was not used in the model, and the largest weight values (both positive and negative) indicate features that are most informative in identifying the component type. This set of features serves as a “signature” for the component type, and the non-zero coefficients were used as input features to a logistic regression to classify novel components, outputting a score between 0 (e.g., “not noise”) and 1 (e.g., “noise”). A threshold applied to the output score provides a binary decision between noise and not noise. For each dataset tested above, we evaluated overall accuracy by receiver operator characteristic (ROC) curve analysis. The ROC curve is a plot that shows a model’s tradeoff between sensitivity (the true positive rate) and 1-specificity (the false positive rate). To generate the ROC curve, we subjected the output of the logistic regression to different thresholds between the values of 0 and 1 in order to calculate a range of sensitivity and specificity values. A threshold closer to 0 means that we label most components as noise (ascribing a label of 1), maximizing the true positive rate (sensitivity), and a threshold closer to 1 means that we label most components as not noise (ascribing a label of 0), maximizing the true negative rate (specificity). We used thresholds between 0 and 1 determined by the best cross validation accuracy (CVA), specificity, sensitivity, and area under this curve (AUC) to assess the overall classifier performance, and calculated confidence intervals for these metrics. The ninety-five percent confidence interval for our results was determined based on the standard error of a binomial. Given k correctly identified networks out of a total of n networks, the observed proportion 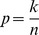 with a standard error:
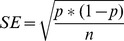
the ninety-five percent confidence interval is 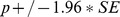.

#### 2.6.6 Comparison with a recently developed method

We tested a recently published method, the Spatially Organized Component Klassifikator (SOCK) with our data to compare the two approaches. SOCK is a method that automatically distinguishes artifacts in ICA decompositions of fMRI, and differs in that classification is based on more “hard-coded” assessments, and so the algorithm does not require any sort of training set [1]. Like our method, the input to SOCK are ICA decompositions performed with FSL’s MELODIC, and this allowed for extending the method to test its ability to predict noisy components in our data. Since the method does not distinguish different kinds of noise, we evaluated it using our data and labels representing “all noise types” for combined individual decompositions (created by combining Data A and Data B) as well as group decompositions (combined group decompositions of Data A and Data B).

### 2.7 Ethics Statement

Data from the Mind Research network were legacy data from a study approved by the Institutional Review Board at the University of New Mexico, and all participants provided written informed consent including a data sharing clause for the MRN Data Exchange. All data were de-identified before use in these analyses. Remaining datasets were publicly available, including (1) the NKI Rockland resource (Nooner et al., 2012), and (2) the MGH-UCLA Human Connectome Project (HCP; Principal Investigators: Bruce Rosen, M.D., Ph.D., Arthur W. Toga, Ph.D., Van J. Weeden, MD). All data were analyzed anonymously.

## Results

### 3.1 Predictive Models for Individual Components

#### 3.1.1 Individual Component Models trained on Data A, tested on Data A: Classifier built using 880 components from healthy control data

The “all noise types” classifier (model M1) was successful in distinguishing noisy components (sensitivity = 0.91, specificity = 0.82, CVA = 0.87, AUC = 0.93) using a subset of 147 features. The features having the greatest weight in the model pertained to regional activation (in gray matter, CSF, and Montreal Neurological Institute (MNI) 152 atlas template edges) and the kurtosis of the time-course. The other classifiers for eyeballs, head motion, and ventricles (models M2, M3, and M4, respectively), were able to accurately identify components of their particular type (specificity = 1.0, 0.99, and 0.99, respectively), but they all had high false negative rates (sensitivity = 0.46, 0.39, 0.43, respectively). Top selected features encompassing subsets of 40, 16, and 5 features for the eyeballs, head-motion, and ventricles models included “percent activation in eyeballs,” “percent total activation in gray matter,” “spatial entropy of the IC distribution,” and the “average distance between local maximum” (eyeballs), “percent total activation in the skull,” “percent total activation in white matter,” “average jump from maximum to minimum intensity,” and “percentage of activation that is left/right symmetric” (head-motion), and “percent total activation in ventricles,” “percent total activation in white matter,” “activation in left caudate,” and “percent total activation in gray matter” (ventricles). The top 10 selected features and weights for each model are included in **Table 2**, and complete results are in **Table S1**.

**Table 2:** “Signatures” of Component Groups.

	All Noise (M1)	Eyeballs (M2)	Head Motion (M3)	Ventricles (M4)
1	Percent total activation in GM	Percent total activation in eyeballs	Percent total activation skull	Percent total activation ventricles
2	Kurtosis of IC distribution	Percent total activation in GM	Percent total activation in WM	Percent total activation in WM
3	Percent total activation MNI152all edges	Spatial Entropy of IC distribution	avg jump max min	Caudate R
4	Percent total activation in CSF	Avg distance btw 10 local max	Percentage activation voxels LR symmetric	Percent total activation in GM
5	Angular L	Max cluster size 10 local max regiongrowing thresh 2.5	Amygdala R	Caudate L
6	Angular R	Mean cluster size 10 local max regiongrowing thresh 2.5	Percent total activation MNI152 all edges	
7	Cingulum Ant R	Percent total activation in WM	power band 0.008 to 0.02 Hz	
8	Temporal Sup L	Skewness of IC distribution	Percent total activation in CSF	
9	Avg distance btw 10 local max	Percentage activation voxels LR symmetric	four lag auto correlation	
10	Temporal Sup R	Percentage activation voxels LR symmetric	Amygdala L	
	**Weights**			
1	0.5586	0.5032	0.549	0.5201
2	0.2665	−0.1894	−0.1565	0.2634
3	−0.2467	0.1884	−0.1077	0.2459
4	−0.2153	0.1797	0.0893	−0.2025
5	0.2098	−0.0674	−0.0711	−0.0674
6	0.1924	−0.0653	0.0571	
7	0.1859	−0.0444	0.0519	
8	0.1637	0.0354	0.051	
9	0.1619	0.0288	0.044	
10	0.1604	−0.017	−0.0425	

Top 10 Chosen Features for classifiers M1, M2, M3, and M4 built using Data A (top), and associated weights (bottom). Gray Matter (GM), white matter (WM), cerebral spinal fluid (CSF). complete set of results included as **Tables S1**.

**Figure 4:**
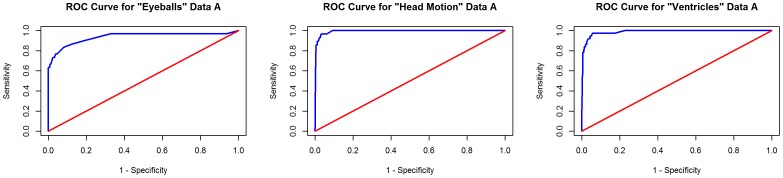
ROC analysis for noise subtype models. ROC analysis of the classifiers trained and tested with ten-fold cross validation on Data A for the “eyeballs,” “head motion,” and “ventricles” models, respectively.

Attempts to build classifiers to distinguish functional networks (primary visual cortex, parieto-occipital cortex, and ventral primary somatosensory cortex) were not successful, performing no better than chance and having no value for prediction. Thus, these models were not extended to other data. **Table 3** shows the classifier model M1 performance tested with 10-fold cross validation with Data A, and **Table 2** shows the top features used in each model. The receiver operator curves, representing the tradeoff between sensitivity and specificity for each model of noise, are displayed in **Figures 3** and **4**. The optimal sensitivity and specificity values reported above for each model represent the point on these curves that maximizes the cross validated accuracies. The red lines on the plots represent a classifier that performs no better than random chance.

**Table 3:** Classifier performance.

	Model		Sensitivity [95% CI]	Specificity [95% CI]	Best CVA [95% CI]	AUC	Features Used	Noise/Not Noise
Noise	M1	All Noise	0.910.88 0.93	0.820.78 0.86	0.870.84 0.89	0.93	147	475/880
	M2	Eyeballs	0.460.25 0.61	1.01.0 1.0	0.980.97 0.99	0.93	40	30/880
	M3	HeadMotion	0.390.21 0.57	0.990.99 1.0	0.970.97 0.98	0.99	16	28/880
	M4	Ventricles	0.430.29 0.62	0.990.99 1.0	0.970.96 0.98	0.93	5	37/880

Performance metrics (sensitivity, specificity, best cross validation accuracy (CVA), area under the curve (AUC)), number of features selected, and proportion of noise components in data for four successful models, including comprehensive noise (All Noise, M1) and three noise subtypes (M2)(M3)(M4), built with and tested with ten -fold cross validation on Data A (healthy control).

#### 3.1.2 Individual Component Models trained on Data A, tested on Data B: Testing models on different data from same institution, same scanner

Four successful models built with Data A (healthy control) were tested to automatically identify noisy ICA components in Data B (638 schizophrenia components from the same institution and the same scanner). The “all noise types” model (model M1) was again successful in distinguishing noisy components (sensitivity = 0.89, specificity = 0.83, CVA = 0.86), and the remaining noise-type component classifiers (models M2, M3, and M4) for eyeballs, head motion, and ventricles, were able to accurately identify noisy components of their particular type (specificity = 1.0, 0.99, and 0.99 respectively). Once again, these classifiers had high false negative rates (sensitivity = 0.56, 0.25, 0.2, respectively). See **Table 4** for classifier performance and **Figure 2** for the receiver operator curve.

**Table 4:** Classifier performance.

	Model		Sensitivity[95% CI]	Specificity[95% CI]	Best CVA[95% CI]	Noise/Not Noise
Noise	M1	All Noise	0.890.86 0.93	0.830.78 0.87	0.860.83 0.88	343 (295)
	M2	Eyeballs	0.560.31 0.80	1.01.0 1.0	0.980.96 0.99	16 (622)
	M3	Head Motion	0.250.05 0.49	0.990.99 1.0	0.980.96 0.99	12 (626)
	M4	Ventricles	0.20.05 0.34	0.990.99 1.0	0.960.94 0.97	30 (608)

Performance metrics (sensitivity, specificity, best cross validation accuracy (CVA), area under the curve (AUC)), and proportion of noise components in data for comprehensive noise (All Noise, M1) and three noise subtypes (M2)(M3)(M4), built with Data A and tested with ten -fold cross validation on Data B (data from the same institution, same scanner, different subject population).

#### 3.1.3 Individual Component Model M1 trained on Data A, tested on Data C, Data D: Testing model M1 on data from different institutions

The “all noise types” model (model M1) built with the 880 components from healthy control was tested to distinguish noisy components for Data C and Data D, which comprise data from (1) a different institution with an equivalent scanner, and (2) a different institution with a different scanner. Model M1 was successful in distinguishing noisy components for a different institution and equivalent scanner (sensitivity = 0.88, 95% CI [0.86 0.90]; specificity = 0.88, CVA = 0.88), and a different institution and different scanner (sensitivity = 0.72, specificity = 0.92, CVA = 0.79). **Table 5** includes M1 performance when tested on Data C, and Data D, and Data B (same institution, same scanner) is also included for comparison. See **Figure 2** for the receiver operator curve for M1 tested on Data C, and for M1 tested on Data D.

**Table 5:** Summary of classifier performance.

Model	Testing Dataset	Sensitivity[95% CI]	Specificity[95% CI]	Best CVA[95% CI]	Noise/Not Noise
M1	**Schizophrenia**Same institution, same scanner	0.890.86 0.93	0.830.78 0.87	0.860.83 0.88	343 (295)
M1	**NKI Rockland Institute**Different institution, same scanner	0.880.86 0.90	0.880.86 0.91	0.880.86 0.89	947 (711)
M1	**Human Connectome Database**Different institution, different scanner	0.720.68 0.77	0.920.88 0.95	0.790.75 0.82	451 (230)

Performance metrics (sensitivity, specificity, and best cross validation accuracy (CVA)) and proportion of noise components in data for model of all comprehensive noise (All Noise, M1) built with Data A and tested with ten -fold cross validation on three novel datasets: Data B (same institution, same scanner, different subject population), Data C (different institution, same scanner), Data D (different institution, different scanner).

### 3.2 Predictive Model for Group Components

#### 3.2.1 Group Component Model M5 trained and tested on combined Data A and Data B: Validation of method with multi-expert standard

To test the method using a standard developed by a cohort of experts, a model to distinguish noisy components “all noise types” (model M5) was built using 44 group components (combined Data A and B). Model M5 was able to successfully distinguish noisy components (sensitivity = 0.91, specificity = 0.81, CVA = 0.87, AUC = 0.82) using a set of 15 spatial and temporal features, detailed in **Table 6**.

**Table 6:** Group Model Performance.

	Noise (Not Noise)	Sensitivity[95% CI]	Specificity[95% CI]	Best CVA[95% CI]	AUC
All Noise	21 (23)	0.910.83 1.0	0.810.75 0.86	0.870.77 0.97	0.82
**All Selected Features**		**Weights**			
Percent total activation in GM		−0.7905			
Dynamic count diff low		−0.5534			
Frontal Inf Tri L		−0.5432			
Temporal Pole Mid L		0.4395			
Kurtosis measure how outlier-prone		0.3655			
Cerebellum Crus1 L		−0.3348			
Insula L		−0.2233			
Angular R		−0.1752			
Cuneus R		−0.1367			
Skewness of IC distribution		0.1189			
Cingulum Mid L		−0.0743			
Occipital Sup R		−0.0314			
Four lag auto correlation		0.0131			
Frontal Med Orb R		−0.0129			
SupraMarginal L		−0.0077			

**“**All Noise types” Group ICA Classifier (M5) (built with combined group ICA decompositions of Data A and Data B) performance, selected features, and weights.

### 3.3 Comparison with Recent Automated Method

The Spatially Organized Component Klassifikator (SOCK) was used to identify artifact for both our individual decompositions (combined Data A and Data B), as well as for combined group components from the same two datasets. To compare with our method, we generated an equivalent model for individual ICA using combined Data A and Data B with the same procedure outlined in 3.1. For classifying artifact in individual ICA, SOCK had moderate performance (sensitivity = 0.52, specificity = 0.89, CVA = 0.69), as compared to our method (sensitivity = 0.91, specificity = 0.89, CVA = 0.90). The low sensitivity is reflective of the method’s high number of false negatives, or not flagging components as noise when they should be flagged. For classifying artifact in group ICA, SOCK again performed moderately, having high specificity and low sensitivity (sensitivity = 0.42, specificity = 0.91, CVA = 0.68) as compared to our approach (sensitivity = 0.91, specificity = 0.81, CVA = 0.87). Complete results are included in **Table 7**.

**Table 7:** Evaluation with other methods.

Method	Component Type	CVA[95% CI]	Sensitivity[95% CI]	Specificity[95% CI]
SOCK Individual	All Noise Types	0.690.65 0.72	0.520.49 0.56	0.890.87 0.91
Our method Individual	All Noise Types	0.900.89 0.91	0.910.88 0.92	0.890.86 0.92
SOCK Group	All Noise Types	0.680.48 0.87	0.420.21 0.64	0.910.79 1.0
Our method Group	All Noise Types	0.870.77 0.97	0.910.83 1.0	0.810.75 0.86

Summary of the evaluation of the Spatially Organized Component Klassifikator (SOCK) tested with our data and “all noise type” labels for individual decompositions (combined Data A and B) and group decompositions (Data A and B), as well as our method’s performance with a new model tested and trained using the same combined Data A and B. Accuracy, sensitivity, specificity, and CVA are reported below.

## Discussion

Though ICA is a powerful technique to identify components of functional connectivity networks, the method is limited since it extracts functional networks as well as noise. Reviewing rsfMRI studies by hand to identify noisy ICA components is problematic in the paradigm of Big Data where there are numerous imaging studies to be analyzed, and approaches that do not employ automatic, intelligent feature selection would be challenging to apply across institutions, scanners, populations, and component types. Our goal was to develop an automated method to identify noisy ICA components to allow automated filtering of rsfMRI data to exclude them prior to data analysis, and we were able to develop an automated classifier that can identify noisy components with reasonable accuracy. Specifically, we built a model that can identify an abstraction of all noise, as well as models to describe specific components related to head motion, eyeball motion, and ventricles. The ability of these models to accurately predict noise using flexible feature selection across different populations, sites, acquisition parameters, and scanner types makes it advantageous to previous work that makes *a-priori* assumptions about the signature of noise.

### 4.1 A Model to Distinguish All-encompassing Noise

The superior performance of models M1 and M5 representing “all noise types” is likely a reflection of the ability of the researcher to have a good sense for what encompasses a component not related to functional networks. Within the “all noise types” component set there are undoubtedly components that the researcher would specify as noise, even if he or she cannot apply a specific label such as “ventricles” or “eyeballs,” and so viewing noise as an abstraction without needing to specify the particular noise type (as is done with the “all noise types” labels) has proven to be a successful strategy for filtering a dataset.

Confidence in the method is raised by assessing the spatial and temporal features selected for each component type. For example, the top selected feature for “all noise types” components between individual and group classifiers is “percent activation in gray matter.” Given that activation in gray matter is a prime feature of a functional network, this result makes sense. The top selected features for noise subtypes included “percent total activation in eyeballs” (eyeballs model), “percent activation in white matter” (head-motion model), and “percent total activation in ventricles” (ventricles model). A particular feature of interest is the highly weighted “dynamic count diff low” from model M5. This feature is the difference between the peak power and minimum power at lower frequencies to the left of the peak. This feature is a derivation of one defined by Allen et. al, namely the “dynamic range” as “the difference between the peak power and minimum power at frequencies to the right of the peak.” This feature was developed to reflect this same idea, but to the left side of the peak. It is likely the case that many noisy components have high power at specific frequencies, and this is reflected by assessing the difference from both the right and the left. However, it is salient that the feature “dynamic count diff high,” the feature defined by Allen et. al reflecting difference between peak power and minimum power to the right of the peak, was not selected. This could be reflective of noisy components having several high counts for higher frequencies, but not for lower frequencies [51]. We find it plausible that our “all noise types” included a substantially larger feature set to achieve equivalent classification performance, as the definition of noise is extended beyond that of one subtype.

This classifier by no means encompasses a domain-wide standard for noise in resting state fMRI, but rather is representative of an individual or group standard that would be desired to filter a larger component set. It is not clear if artifact selection should be handled prior to or following group ICA, however the method is useful in either case. Our method would greatly accelerate the data review process currently performed by researchers, even if researchers want to review the automated results for accuracy.

### 4.2 Models to Distinguish Specific Noise Types

The top chosen features for each of the component classifiers encompass both spatial and temporal features, suggesting the importance of both. We believe that the high false negative rate for specific noise types (models M2, M3, and M4) can be attributed to the idea that different types of noise often are mixed in the same component. For example, when testing model M2 (eyeballs), 11 components that were identified by the researcher as eyeballs were missed by the classifier. More careful inspection of these components revealed that, while they did have obvious signal in the eyeballs, there was additional signal in other parts of the brain. Given that the model weights the feature “percent activation in eyeballs” highly (0.5032), a component with eyeballs activation might be missed if it has activation in these other areas. This observation is a good rationale for the approach taken by Allen et. al, for which he ascribed components with the labels “good,” “bad,” and “mixed” [51]. However, in these cases of a high level of false negatives, it is notable that there were no false positives, meaning that for our example, the labeled components that were tagged as eyeballs were in fact eyeballs. In practice, this means that we could use the model to confidently remove strong eyeball components and reduce the size of the remaining subset to be reviewed by the researcher. We have shown that the model is flexible enough to perform well with an abstraction of all noise, and for specific noise types.

### 4.3 Comparison with Recent Automated Approach

We believe that the SOCK algorithm’s high false negative rate (missing noisy components) is due to the algorithm’s “hard-coded” approach, meaning its inability to learn the noise signature of a dataset. While the specificity is high (meaning that components flagged as artifact indeed were likely to be artifact), many noisy components were unfortunately missed. More careful investigation of these components revealed many spatial maps with mostly voxels for eyeball activation, suggesting that the algorithm may benefit from a feature that is based on a mask of this area, which may not be included in the “edge activity measure” that the algorithm does consider. The algorithm missed many noisy components with temporal signatures limited to a narrow frequency range across time-points, given that the range did not exceed 0.08 Hz. The lower sensitivity might also be explained by the SOCK method’s aim to not eliminate any components that could be of neuronal origin when there is a mixture of noise and biological signal. This rejection criterion differs from our approach that aims to maximize overall accuracy. We believe that it can be risky to make a-priori assumptions about noise; however testing this method gave us confidence in the approach’s sensitivity, or that the components flagged as artifact are likely to be artifact. The discrepancy between our analysis and the SOCK algorithm finding a small number of discordant ICs in the original paper is likely attributable to the different data used in both cases. We were unable to evaluate the approach with any sort of ROC curve analysis because the output of SOCK is a binary decision indicating artifact or not. We report combined evaluation metrics for combined individual ICA for both Data A and B because we saw equivalent performance of the algorithm when the data were separated.

### 4.4 Independent Component Analysis

While there are many modifications of the general decomposition algorithm to perform independent component analysis [46], [47], [59], we used fastICA in our work because it is a solid, practical approach that is commonly used with the MELODIC toolbox in FSL. While it is arguably challenging to compare components between individuals, the Z score maps were chosen for comparison because they normalize each component data, reflecting comparable degrees of activation from the individual-specific means. Using Z score maps, extracted temporal features can be used to compare overall levels and changes in these levels, meaning that for the networks in any two particular individuals, we are not comparing the values themselves, but rather the relative strength of the networks. Our choosing to threshold the maps to include only the highest and lowest activation voxels was arbitrary, and based on the idea that not thresholding the maps would mean including activation that is not statistically significant in many more spatial areas. Thresholding the maps emphasizes regions with the most contribution to the component. Additional activation from not thresholding the maps would likely lower the variance for the voxel count of regional spatial features, and we speculate that this change would make it harder for the classifier to find uniqueness between the components. This hypothesis has not been formally evaluated. In the case of looking for differences in networks between groups it could be the case that more meaningful network differences are found at subtle differences at the edges of these maps, and we see this as an area for future work. However, for this work to identify noisy components, we believe that a thresholding strategy that favors more sparse spatial maps is favorable.

### 4.5 Regional, Abstracted Spatial and Temporal Features

In demonstrating that it is possible to characterize noisy components based on higher level spatial and temporal features over standard voxel-wise approaches, this work is a strong proponent for the development of more abstract methods to make inferences over large data. We believe that voxel counts for each regional spatial feature (regions and tissue-types) represent the component map abstractly, and this abstraction can better group different component types across large data. For example, while components pertaining to head motion may not overlap perfectly on a voxel-wise level, components of these types will have voxels that are generally grouped around the brain, and so this similarity is better reflected in a regional voxel count. We used the AAL atlas to define these regional voxel counts because it provides a resolution that can abstract to generalized anatomical regions. Using individual voxel activations as features would have the potential to over-fit the data, and further, would drastically increase the number of features to hundreds of thousands.

A huge benefit of a set of regional features defined by a standard atlas means that they can be ascribed with anatomical labels to allow for easy human interpretability, which is not always true of features that have been used to distinguish noisy components. Beckmann recently explored the possibility of using the spatial and temporal nature of components to detect different kinds of noise [22], and Thomas et.al suggested that features of the power spectral density of the components could be valuable for identifying “structured noise” (regular, non-neurological signal like respiration and cardiac) and “white noise” (unstructured noise) [52]. Perlbarg defined subject-specific respiratory and cardiac noise signatures as time-courses isolated to ventricles, brainstem, and arteries, and characterized structured noise in the rest of the brain by correlating components with these signatures [60]. While these approaches can be used to filter a set of components, classification based on comparing similarity of time-courses and spectral features is less humanly interpretable than a clear list of features and associated weights.

We believe that a robust set of abstract features with an adaptive algorithm is also strong in that it does not make assumptions about the noise signature of a particular dataset. Recent work to automatically identify noisy components makes these assumptions, assuming that a particular spatial location or temporal frequency is always true of noise [1], or that noise follows a static pattern in the time-course alone [34]. While these methods work well when the noise in the signal has an expected pattern, these methods do not allow for noise that deviates from this expectation, do not support identifying the signature of specific noise types, and could not be extended to learning a signature for a novel kind of component. We strongly believe that a solid approach should allow for the flexibility of selecting small subsets of features from a larger set to best define different types of noise.

### 4.6 Limitations

Our work has several limitations. First, we recognize that it is challenging to automatically recognize all kinds of noise and that the quality of our automatic classification approach is limited by the case data we used to develop our model. The determination of noisy ICA components could vary among readers; however we are not aiming to establish an all-encompassing classifier for noise in resting fMRI, but rather to demonstrate that different component types carry computational signatures which can be identified using an automated approach combined with intelligent feature selection. Perhaps more important than the classifier itself is discovery of the types of features that abstractly define noise and which can be used to create automated, robust filtering pipelines for the ever increasing amount of data becoming publicly available. While there is likely to be variance in values based on acquisition protocol, preprocessing and analysis parameters, and subject population, we will likely see the same types of noise across many datasets. For example, motion-related artifact will likely have a substantial amount of signal in the skull and surrounding tissue. While researchers studying schizophrenia in children would likely have more substantial motion artifact in their data than an equivalent adult study, the researchers could employ these methods to create customized filtering pipelines for their particular datasets, and further, identify components that share a computational signature of noise that would be difficult to identify without these methods.

A second limitation is that our method requires choosing a threshold for separating noisy from non-noisy ICA components. While we chose to operate at the point that maximizes overall accuracy, the choice of where to operate on the receiver operator curve may vary depending on the goals of the researcher. Minimizing false positives (identifying a component as noise when it is not) would be achieved by maximizing specificity. This choice would ensure that only components that are most likely to be noise are filtered. The researcher could review all putative noisy ICA components to make a final decision. In this scenario, the researcher still receives benefit from the method, since a portion of the cases need not be reviewed.

A third limitation is that component spatial maps and time courses are influenced by order selection. Automatic order selection is ideal for our application because it estimates order from the data, meaning that more artifacts will lead to a higher order, however a downside is that it leads to a comparison of different orders between subjects. While this may be a point of discomfort for some researchers, we believe that an automatic estimation is ideal because it cannot be assumed that datasets share equal biological or artifactual complexity. Additionally, the different orders are comparable in our feature space as each component is Z-transformed by subtracting the mean and dividing by the standard distribution. The resulting voxel values are “Z-scores,” and each Z-score map is thresholded to include the 5% of voxels most strongly exhibiting the extracted signal. Another area of concern is related to the possibility that the method could have varying performance with components extracted from images of varying resolution. It is notable that although the automatic order estimation resulted in a larger number of components extracted for the higher resolution data C, the model performs equivalently well.

A fourth limitation is that we used a small number of datasets. We strongly believe in evaluation of our approach across more data, and this is reflected in our choice to publicly release our software (http://www.vbmis.com/bmi/noisecloud) and include results for data that is publicly available to other researchers (Data C and Data D). We expect that our statistical-based classification methodology would be extensible and generalizable to other datasets, though feature weightings will likely be adjusted during model creation to optimize performance. Within each individual ICA decomposition we speculate that there are distinct probabilities for seeing each component type, and so further work might take these component type frequencies into consideration, possibly allowing for more probabilistic approaches applied to classifying the data.

A fifth limitation is that a metric of confidence was not obtained during the creation of our standard labels. Neuroscience laboratories often manually filter components based on visual inspection of the data alone, and so our approach to developing a gold standard of noisy ICA components mirrors that practice.

A final limitation is that although we attempted to build models to distinguish functional networks, many of our features were developed based on artifacts in rsfMRI, and we suspect this might be why we were not successful in finding patterns of features to predict specific functional networks. Additionally, we recognize that even spatially consistent functional networks can be split into an anterior and posterior portion, making the label creation problematic. The manual annotation was done to select components that were most strongly indicative of a particular noise or network subtype, missing components that when put together might comprise the same functional network. This could be another strong reason that we were not successful in building a classifier to predict any specific functional network. The variability of components speaks to approaches that filter or manipulate component data before attempting classification [36]. It would be interesting to extend our feature set to include features that would better define functional networks, as well as introducing a “mixed” label type that allows for components with elements of noise and functional activation. We believe that more successfully classifying functional components could be possible using this approach, provided a suitable training set is acquired.

We believe that our approach is extendible beyond the domain of distinguishing noise from functional networks. For example, the approach would be flexible to non-traditional needs such as finding networks with mirror-like qualities, given a suitable training set, or creating a computational signature for other types of brain maps. However the method may not perform well in the case of gross brain pathology, as many of the spatial features are based on standard atlas regions that assume spatial consistency between individuals. In the case that a meaningful spatial or temporal metric is not currently in the database, the method’s ability to select a sparse set of features from a very large set makes addition of this metric non-detrimental to the method’s performance. Our infrastructure that stores features in a database, for query at runtime, also means that adding a feature is easy to accomplish. We find the possibility of researchers exploring different component types and contributing to the database to be exciting, and look forward to further work in exploring the creation of new standards and features that might better classify functional and other types of brain maps.

## Conclusion

This work demonstrates that noisy ICA components can be recognized using an automated classification method which we developed. We show it is possible to accurately predict different kinds of noisy ICA components using intelligent feature selection paired with an automated method. This may have utility to neuroscience researchers to automatically filter out noise components as they analyze large publicly available functional neuroimaging datasets. While there will likely continue to be controversy about what constitutes a particular functional network, our work demonstrates that it is possible to computationally represent a researcher’s or group’s labeling of components across different datasets to identify components that match that standard. We believe that our methods are generalizable and could be extended to automated recognition and classification of other types of brain maps beyond noisy components. Enabling researchers to use our method with their data to more quickly and automatically identify functional networks vs. noise components could greatly enable pursuing data-driven methods for identifying brain-based biomarkers of neuropsychiatric disorders in large scale neuroimaging datasets [61].

## Supporting Information

Table S1 **Selected Features.** Spatial and temporal features and associated weights for each of the successful individual ICA models for comprehensive noise (M1) and three noise subtypes (M2)(M3)(M4), built with Data A, as well as the group ICA “All Noise types” model (M5). A weight of zero indicates that the feature was not selected in the model.
(XLSX)

Table S2 **Spatial and Temporal Features.** Names, descriptions, and links to scripts for all spatial and temporal features used to build the models.
(XLSX)
